# Computational analysis of the *AP2/ERF family* in crops genome

**DOI:** 10.1186/s12864-024-09970-0

**Published:** 2024-01-23

**Authors:** Shouhartha Choudhury

**Affiliations:** 1 Har Gobind Khorana School of Life Sciences, Assam University, Silchar-788011 Assam, India; 2 Department of Biotechnology, Assam University, Silchar-788011 Assam, India; 3 Department of Life Science and Bioinformatics, Assam University, Silchar-788011 Assam, India

**Keywords:** *AP2/ERF superfamily*, *ERF/DREB family*, Abiotic stress and development

## Abstract

**Background:**

The *Apetala 2/ethylene-responsive factor family* has diverse functions that enhance development and torment resistance in the plant genome. In variation, the *ethylene-responsive factor (ERF) family* of TF’s genes is extensive in the crop genome. Generally, the plant-specific ethylene-responsive factor family may divided by the *dehydration-responsive element-binding (DREB) subfamily*. So, the *AP2/ERF super-family* demonstrated the repeated *AP2 domain* during growth. The sole *AP2 domain* function represents abiotic stress resistance. Also, the *AP2 with B3 domain* enhances during the replication of brassinosteroid.

**Objective:**

The study objective is to investigate the *Apetala 2/ethylene-responsive factor* family in a model organism of the *Arabidopsis thaliana* for comparative analysis towards *Solanum lycopersicum* (Tomato), *Brassica juncea* (Indian and Chinese mustard), *Zea mays* L. (Maize) and *Oryza sativa* (Indian and Japanese Rice). So, examinations of the large *AP2/ERF super-family* are mandatory to explore the *Apetala 2 (AP2) family, ERF family, DREB subfamily,* and *RAV family* involved during growth and abiotic stress stimuli in crops.

**Methods:**

Therefore, perform bioinformatics and computational methods to the current knowledge of the *Apetala 2/ethylene-responsive factor* family and their subfamilies in the crop genome. This method may be valuable for functional analysis of particular genes and their families in the plant genome.

**Results:**

Observation data provided evidence of the *Apetala 2/ethylene-responsive factor (AP2/ERF) super-family* and their *sub-family* present in *Arabidopsis thaliana* (Dicots) and compared with *Solanum lycopersicum* (Dicots), *Brassica juncea* (Dicots), *Zea mays L.* (Monocots) and *Oryza sativa* (Monocots). Also, remarks genes in *Oryza sativa*. This report upgraded the *Apetala 2/ethylene-responsive factor (AP2/ERF)* family in the crop genome. So, the analysis documented the conserved domain, motifs, and phylogenetic tree towards Dicots and Monocots species. Those outcomes will be valuable for future studies of the defensive *Apetala 2/ethylene-responsive factor* family in crops.

**Conclusion:**

Therefore, the study concluded that the several species-specific TF genes in the *Apetala 2/ethylene-responsive factor (AP2/ERF)* family in *Arabidopsis thaliana* and compared with crop-species of *Solanum lycopersicum*, *Brassica juncea*, *Zea mays L.* and *Oryza sativa*. Those plant-specific genes regulate during growth and abiotic stress control in plants.

## Highlights

• The study is significantly associated with crop development.

• The developmental and abiotic stress-responsible gene families observed in different crops.

• The *AP2 family* originated during the growing embryo, leaf, and flower.

• The *ERF/DREB families* initiated abiotic stress such as freezing, drought, salt, low oxygen, oxidative, osmotic, heat, heavy metals, and soil salinity.

• Finally, the *RAV family* implicated the solution of brassinosteroid.

• So, the analysis data provided valuable information for the Department of Agriculture Biotechnology.

• Also, the documented data are valuable for the plant database and research.

## Introduction

The plant-specific *Apetala 2/ethylene-responsive factor family* genes are predominant in the dicotyledonous and monocotyledonous plant genomes. Also, the *AP2/ERF (Apetala 2/ethylene-responsive factor) family* is typically divided into *subfamilies*: (1) The primary *AP2 family*, (2) the *ERF/DREB family*, and the last (3) *RAV family*. The *AP2 family* illustrated the duplex *AP2 domain* associated with developmental processes like growing embryos, leaves, and flowers in plants. The classical *ERF family* proposed the sole *AP2 domain* plays a core function in the *Apetala 2/ethylene-responsive factor (AP2/ERF) family*. So, the defensive *ERF family* and stress-responsive *DREB subfamily* functioned as abiotic stress controls such as freezing, drought, salt, low oxygen, oxidative, osmotic, heat, ABA ethylene, jasmonic acid, and abscisic acids have major upshot on the growth and production of plants. The third *RAV family* proposed a combined function of the *AP2* with the *B3 domain* involved during the reaction of brassinosteroid in the plant genome. Those identified twice *Apetala 2 domain*, sole *Apetala 2 domain*, and combined between *Apetala 2* with *B3 domain* consist of amino acid residues involved in DNA binding [1]. The first *AP2 (Apetala 2) domain* is reports in a model organism of *Arabidopsis thaliana*. Recent empirical data illustrated the repeated *AP2 domain* regulates during the developmental processes in crop variety (i.e. flower, meristem, leaf, and embryo development) [2–6]. On the other hand, the ethylene-responsive factor (*ERF*) family divides into two main subfamilies: *C-repeat/dehydration-responsive element binding factors family (CBF/DREB family)* [7–9]. The plant-specific *AP2 domain* observed as a conserved DNA-binding domain called e*thylene-responsive element binding factors* or *ERFs* (i.e. *ERF1*, *ERF2, ERF3*, and *ERF4*) generally binds to the GCC box motifs [10–12]. However, the major *Apetala 2/ethylene-responsive factor (AP2/ERF) family* implicates diverse functions like hormonal signal transduction, cellular processes, regulation of metabolism, and growth processes in plants [6, 8, 13–23]. In December, 2000, the Arabidopsis Genome Initiative (AGI) sequenced the genome of a model plant called *Arabidopsis thaliana* and identified 145 genes in the broad *AP2/ERF family* [8]. Also, particular genes in the supreme *AP2/ERF family* require to determine again. So, we can observe the likelihood of *AP2 domain*-mediated genes play a role and physiological aspect in plant species. Also, a transgenic experiment will be necessary to govern the biological phenomenon of a particular gene in the defensive *AP2/ERF family* in plant genomes. The previous evolutionary study shows that the large *Apetala 2/ethylene-responsive factor families* classified into subfamilies are closely related [24–26]. A comparative and functional study of the particular gene from the *AP2/ERF (Apetala 2/ethylene-responsive factor) family* in crop-specific *Arabidopsis thaliana (*Dicots)*, Solanum lycopersicum (*Dicots), *Brassica juncea (*Dicots), *Zea mays L. (*Monocots) and *Oryza sativa (*Monocots) is necessary for functional abundance. This process and evaluation of the link between gene families would provide a significant direction for predicting and upgrading the species-specific *transcription factor* genes in the particular genome. The current availability of the draft genome sequences of *Arabidopsis thaliana, Solanum lycopersicum*, *Brassica juncea*, *Zea mays L.,* and *Oryza sativa* allowed comparative and functional analysis between plant genomes, which is valuable for the practical and evolutionary diversity of gene families in the genome. In this work, an establishment and comprehensive investigation of the *Apetala 2/ethylene-responsive factor family* in *Arabidopsis thaliana, Solanum lycopersicum*, *Brassica juncea*, *Zea mays L.* and *Oryza sativa* attempts. Also, the genes in the *Apetala 2/ethylene-responsive factor family* in the *Arabidopsis thaliana* genome was survey again and also compared with crops of *Solanum lycopersicum*, *Brassica juncea*, *Zea mays L*. and *Oryza sativa*. A comparative and functional study between Dicots (*Arabidopsis thaliana*, *Solanum lycopersicum*, *Brassica juncea*) and Monocots (*Zea mays L.* and *Oryza sativa*) perform. So, the study reviewed the comparative and functional genomics of the *Apetala 2/ethylene-responsive factor family* response to growth and abiotic stress response in plants.

## Results

The primary sequence demonstrated the formation of nucleotides and peptides in the *ERF109 (RRTF1)* gene in *Arabidopsis thaliana*. The sequence composed of 1386 nucleotides and 268 peptides among 64 peptides bind to the DNA sequence called *AP2 domain* (Table 1).

**Table 1:** Query sequence

> ERF109 AAACACAAACAAAACTCATATTTTCAATCTCCAGGTGCTTTACACCAACAGAGTCGCAAGAAAACAAAAACCAAACTCGGATTTAGTTTGACAGAAGAAGGAATCGAGAGTCGGGTATGCATTATCCTAACAACAGAACCGAATTCGTCGGAGCTCCAGCCCCAACCCGGTATCAAAAGGAGCAGTTGTCACCGGAGCAAGAGCTTTCAGTTATTGTCTCTGCTTTGCAACACGTGATCTCAGGGGAAAACGAAACGGCGCCGTGTCAGGGTTTTTCCAGTGACAGCACAGTGATAAGCGCGGGAATGCCTCGGTTGGATTCAGACACTTGTCAAGTCTGTAGGATCGAAGGATGTCTCGGCTGTAACTACTTTTTCGCGCCAAATCAGAGAATTGAAAAGAATCATCAACAAGAAGAAGAGATTACTAGTAGTAGTAACAGAAGAAGAGAGAGCTCTCCCGTGGCGAAGAAAGCGGAAGGTGGCGGGAAAATCAGGAAGAGGAAGAACAAGAAGAATGGTTACAGAGGAGTTAGGCAAAGACCTTGGGGAAAATTTGCAGCTGAGATCAGAGATCCTAAAAGAGCCACACGTGTTTGGCTTGGTACTTTCGAAACCGCCGAAGATGCGGCTCGAGCTTATGATCGAGCCGCGATTGGATTCCGTGGGCCAAGGGCTAAACTCAACTTCCCCTTTGTGGATTACACGTCTTCAGTTTCATCTCCTGTTGCTGCTGATGATATAGGAGCAAAGGCAAGTGCAAGCGCCAGTGTGAGCGCCACAGATTCAGTTGAAGCAGAGCAATGGAACGGAGGAGGAGGGGATTGCAATATGGAGGAGTGGATGAATATGATGATGATGATGGATTTTGGGAATGGAGATTCTTCAGATTCAGGAAATACAATTGCTGATATGTTCCAGTGATAAATGAGCTCTTTCTTGTTGGCGTTTTTTGGAGTTAAGTGCAAGAAGAGATTGACACTGTGGCTTGTTTAAAGTGAACAAGAACAAGAAAGCATGTAATTAGTAGTCTCATTCTTTTGTTTGTGGTCAATTCTATGTTTATCTCATATAAAATCTGAGTTAAACCTATCTGAGGAGAGAGTAAATAAAGAGGTTAAGAAACCCAACATTGGTCTGAATTATAAACGTAAGTGTCAACGTTGTTTATAAAGGAGAAAACTATAATTGGTGACAAAAGACATAAAGAAAAGATGTCTACTCCTACAAAGCATCGCGTGCAGCTATTCGACAAACAATGGCATCTCCCAGAGAGGAAATTCCGAGCTCTTGGCTAGTTATCTTGTAATGCTGAAAACATGAATGTATTTGAGTTTATTTCTGTAACATTGGAAGCGAAATAAAAGGGTTATCAACTGTTACCAA
> ERF109 MHYPNNRTEFVGAPAPTRYQKEQLSPEQELSVIVSALQHVISGENETAPCQGFSSDSTVISAGMPRLDSDTCQVCRIEGCLGCNYFFAPNQRIEKNHQQEEEITSSSNRRRESSPVAKKAEGGGKIRKRKNKKNGYRGVRQRPWGKFAAEIRDPKRATRVWLGTFETAEDAARAYDRAAIGFRGPRAKLNFPFVDYTSSVSSPVAADDIGANASASASVSATDSVEAEQWNGGGEDCNMEEWMNMMMMMDFGNGDSSDSGNTIADMFQ

Query gene: (a) Nucleotide and (b) Peptide

So, take a closer look at the plant-specific *AP2/ERF family* and analyze genes known so far; those have different composition and functional domains. Also, the observation summarized the total number of *Apetala 2 domains* in a model organism of *Arabidopsis thaliana* and compared it with crops of *Solanum lycopersicum, Brassica juncea, Zea mays L.,* and *Oryza sativa* (Table 2).

**Table 2:** Summary of the *AP2 domain* and *RRTF1*

Species	HMMER	BLAST2	BLAST2GO
*Arabidopsis thaliana (Dicots)*	224	141	1
*Solanum lycopersicum (Dicots)*	182	129	2
*Brassica juncea (Dicots)*	470	391	6
*Zea mays L. (Monocots)*	327	187	2
*Oryza sativa (Monocots)*	178	108	1

Summary of the algorithm hits in all species

**Fig. 1:**
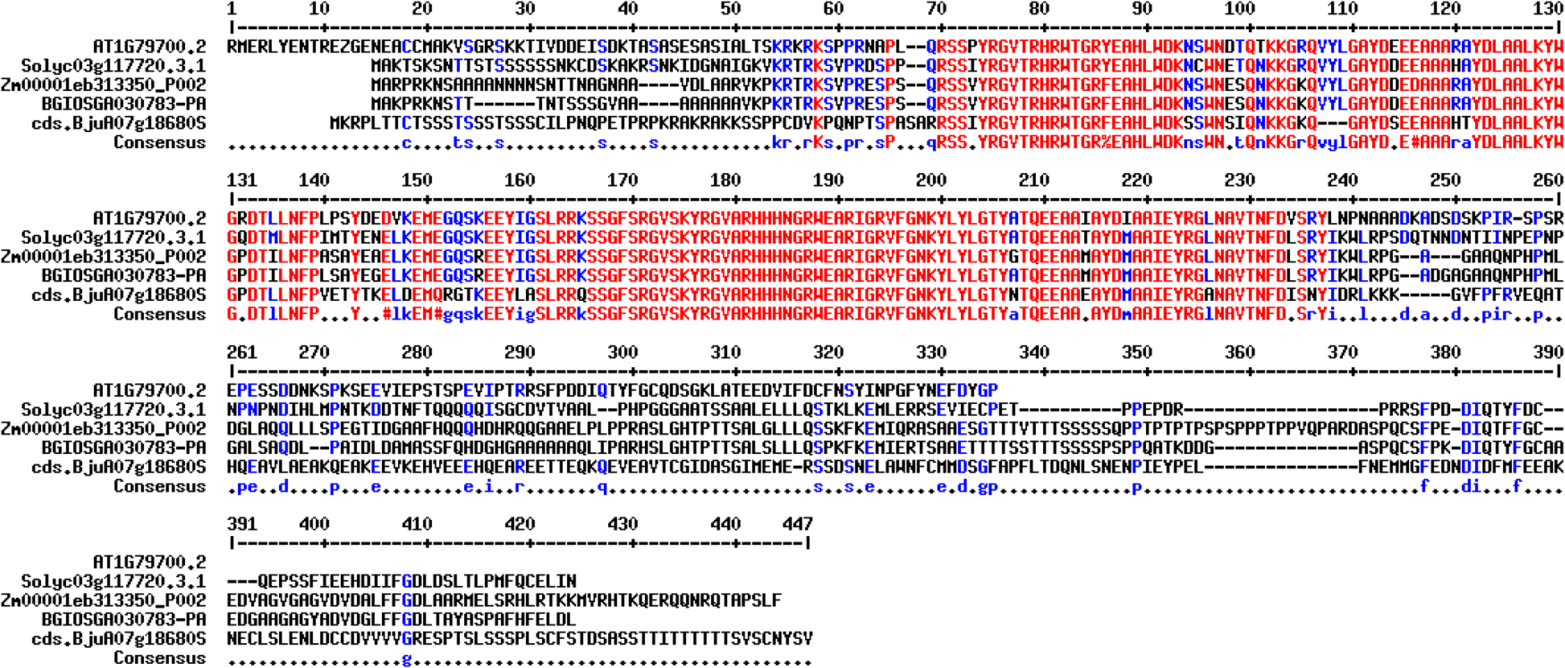
Conserved repeated *AP2 domain* in the *AP2 family*

**Fig. 2:**
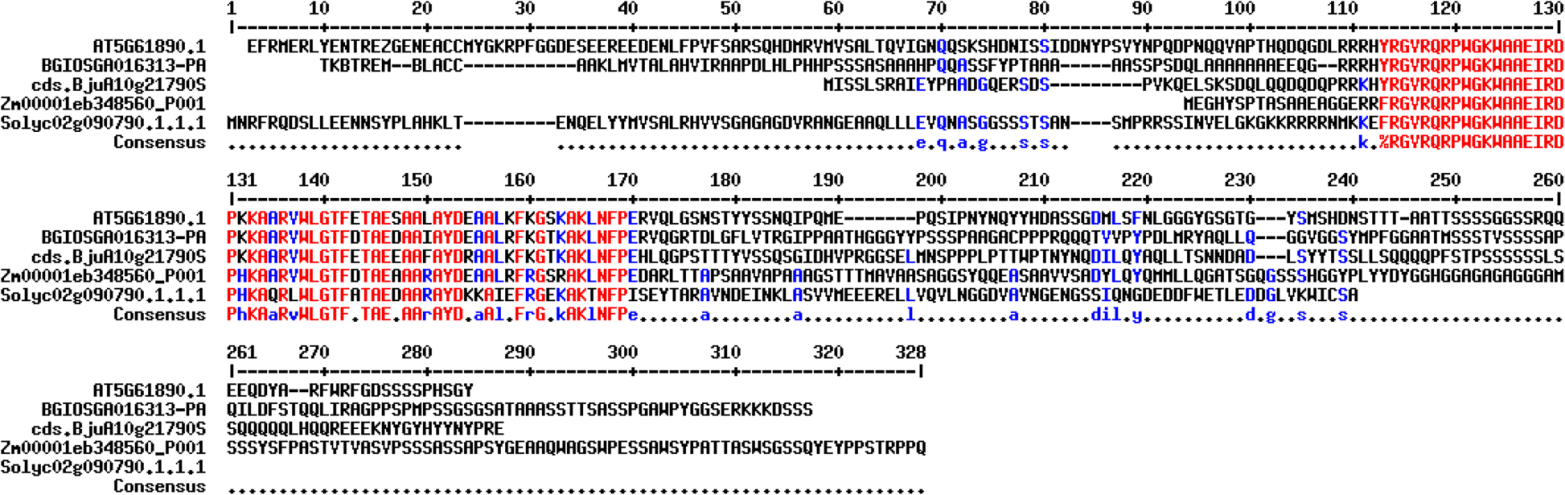
Conserved single *AP2 domain* in the *ERF family*

**Fig. 3:**
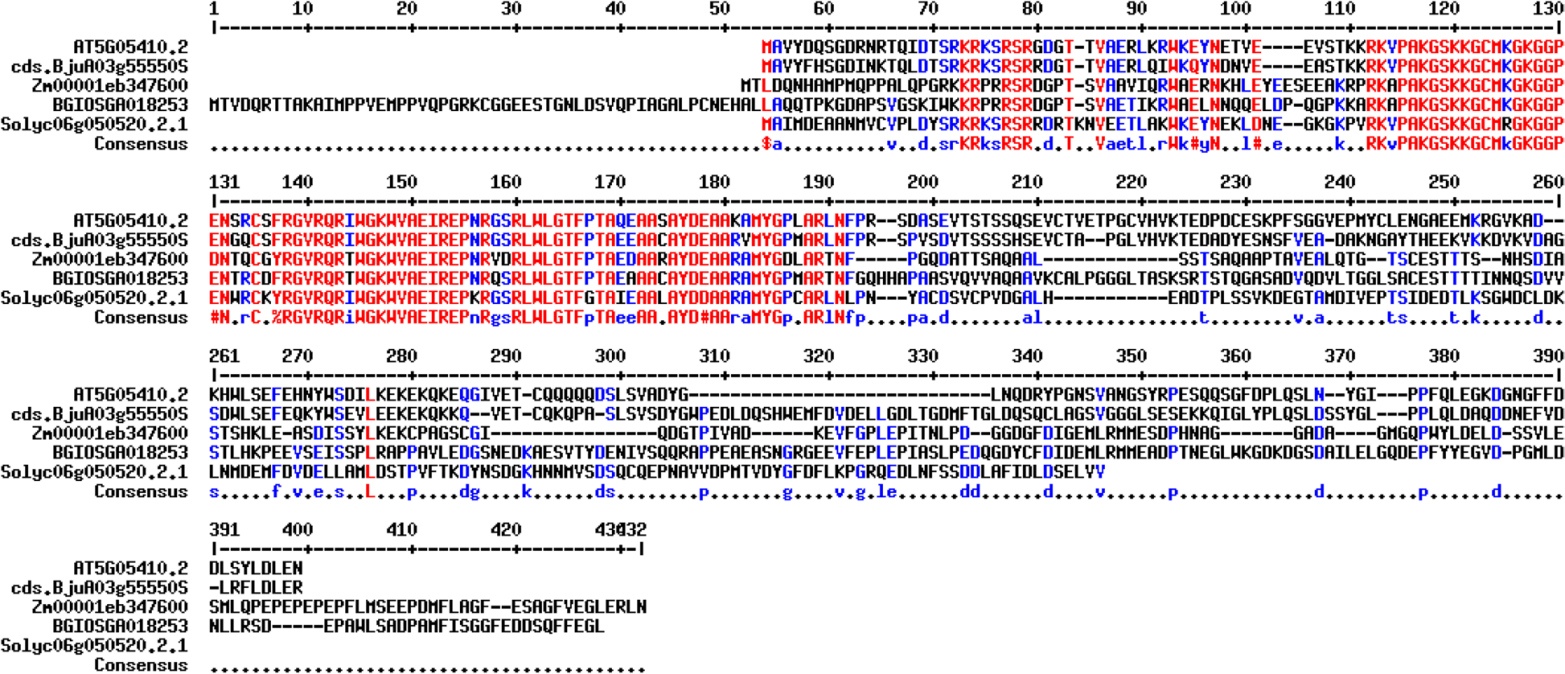
Conserved single *AP2 domain* in the *DREB subfamily*

**Fig. 4:**
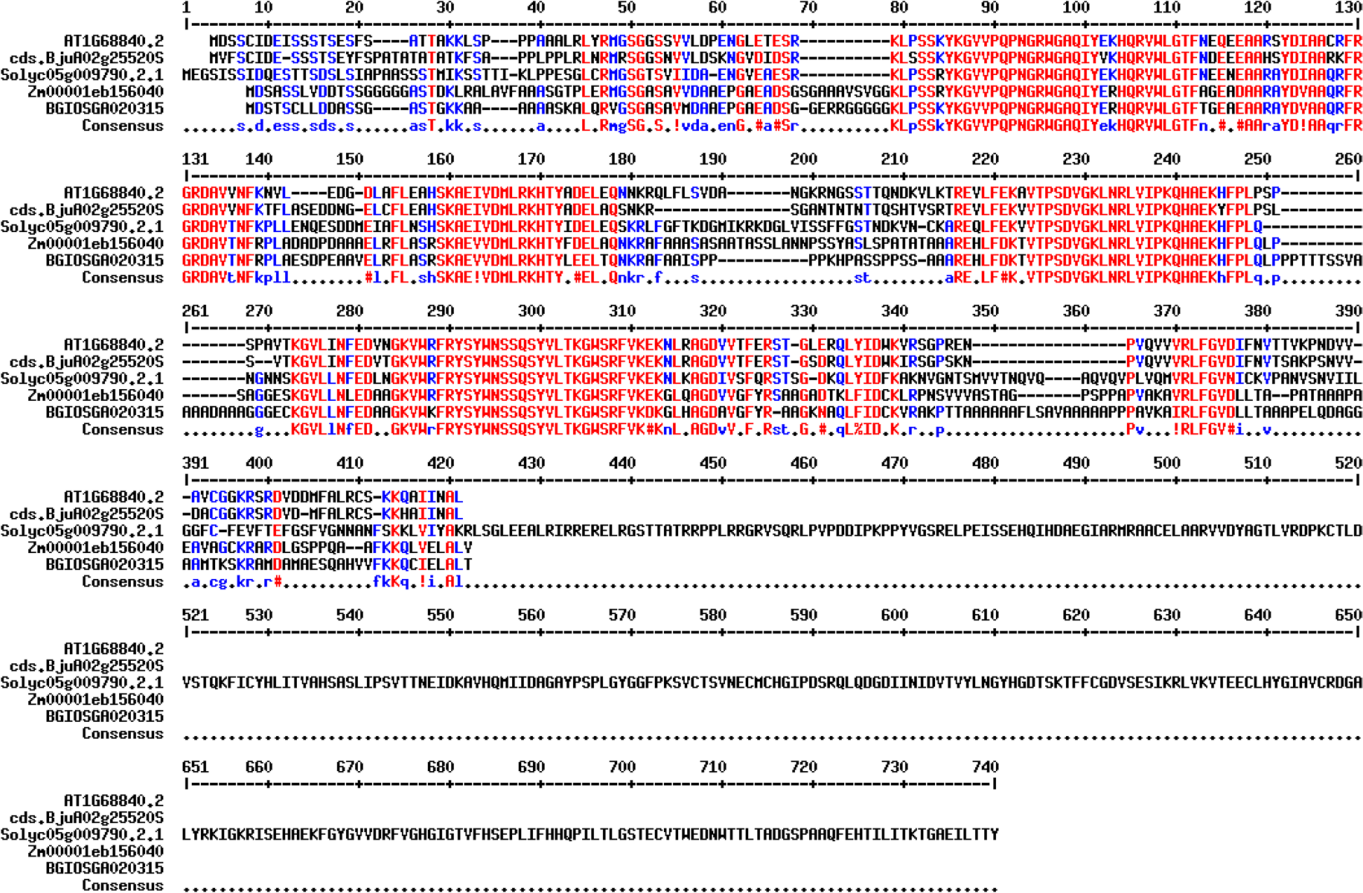
Conserved *AP2 *with *B3 domain* in the *RAV family*

**Fig. 5:**
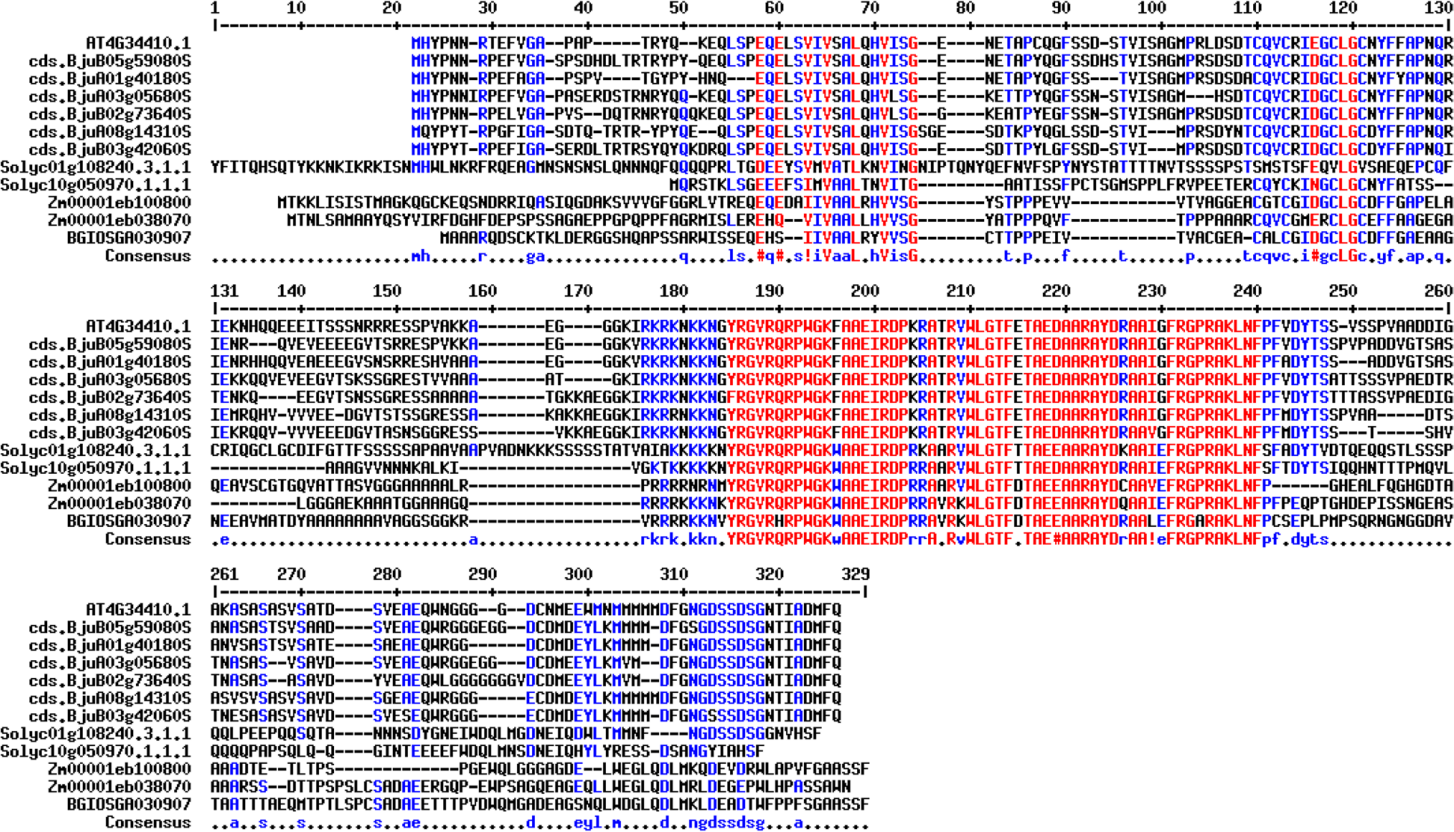
Conserved *AP2 domain* in *RRTF1*

**Fig. 6:**
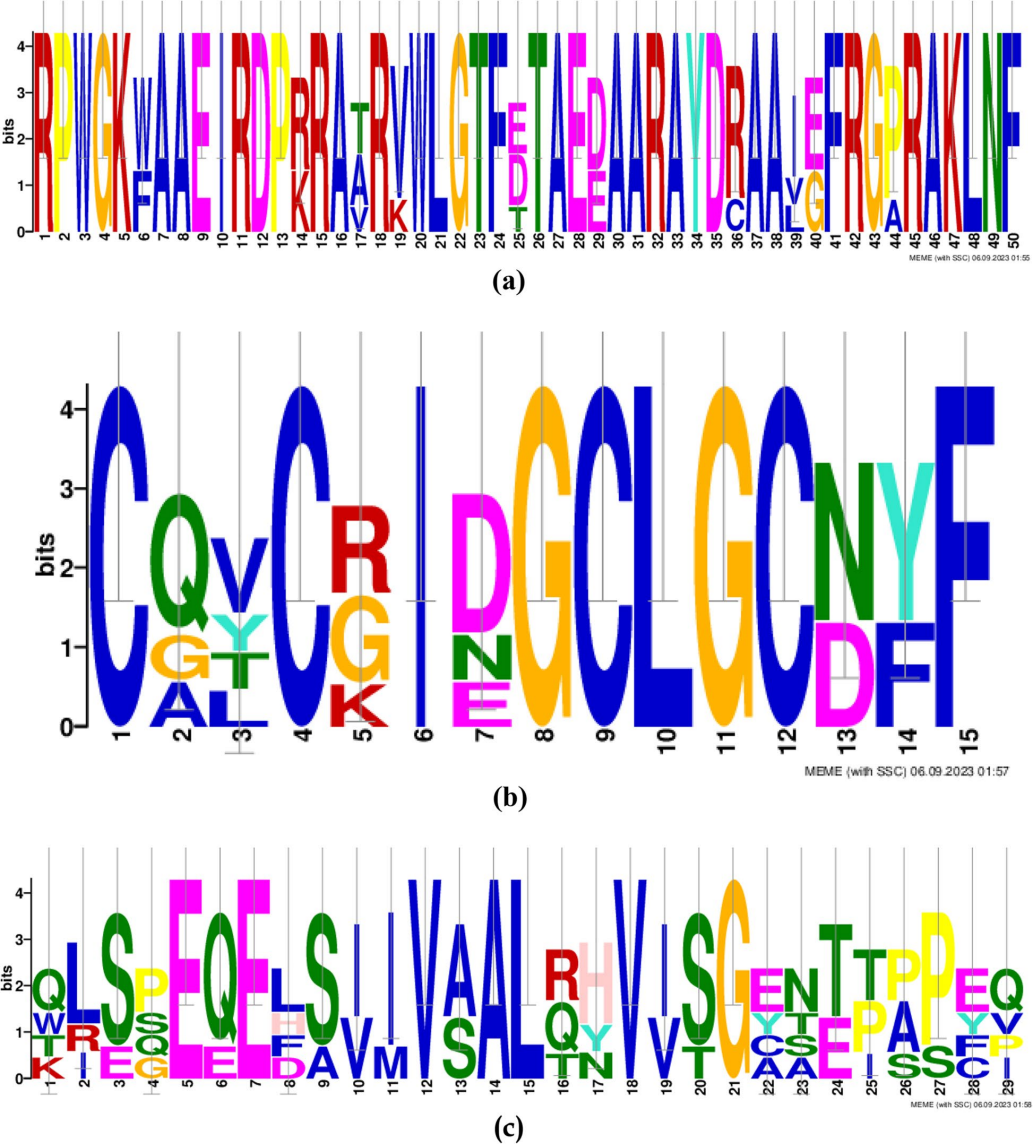
Sequence motifs in *RRTF1* (**a**, **b**, and **c**)

**Fig. 7:**
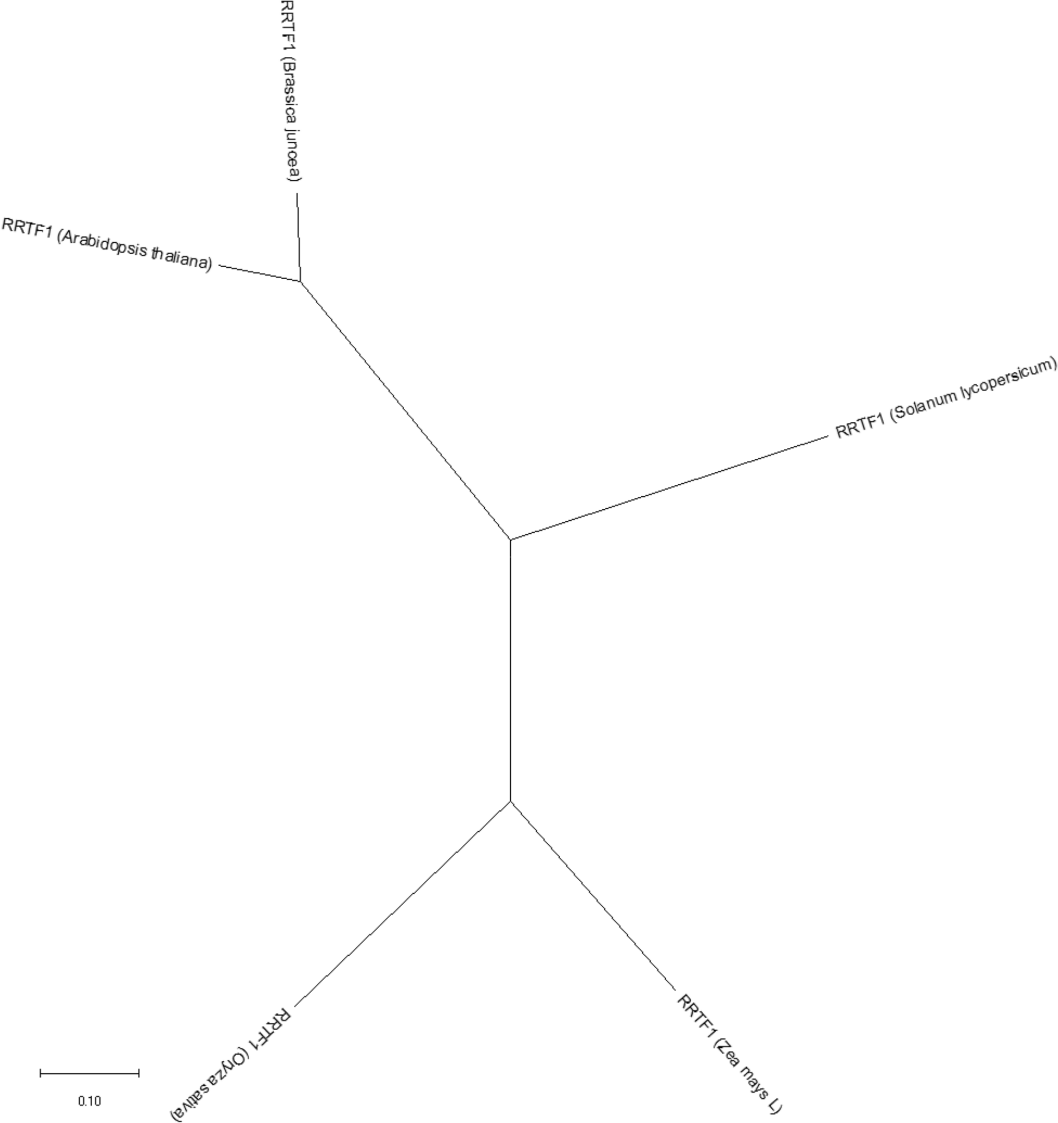
The molecular evolutionary link of the *RRTF1* among all species

**Fig. 8:**
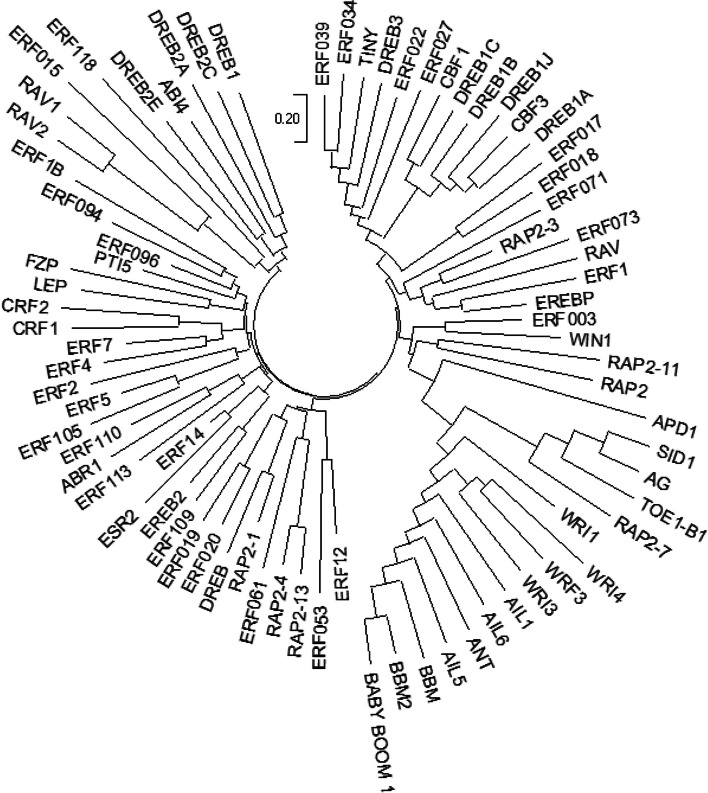
Evolutionary link between genes in the *AP2/ERF family*

Also, the gene ontology (GO) annotation demonstrated the sequence accuracy of the *RRTF1 (ERF109)* gene in the defensive *AP2/ERF family* in all species (Table 3). Further, the GO annotation of the *RRTF1* gene demonstrated the molecular function, cellular component, and biological process in particular organisms. Also, remark genes in *Oryza sativa* (Indian Rice): Os02g42580, Os02g52880, Os03g02650, Os04g36640, Os04g48330, Os06g42910 and Os12g07030. The observed gene in the crop of *Oryza sativa*: LOC_Os06g09717.1 was completely identical with OsERF#139 (Os06g09730) and OsERF#010 (Os06g09690), afterward proposed a new gene Id: Os06g09717. So, the crop-specific transcription factor data analysis documented the total *AP2 domai*n-mediated isoforms in the *AP2 family*, *ERF family*, *DREB subfamily*, and *RAV family* between *Arabidopsis thaliana, Solanum lycopersicum, Brassica juncea, Zea mays L.* and *Oryza sativa* accordingly. Also, the multiple hits of repeated *AP2 domain*, *single AP2 domain*, and *B3 domain* are listed from all species for sequence alignment. The MSA demonstrated the high censuses (90%) sequence is conserved in the *AP2 family*, *ERF family*, *DREB subfamily*, and *RAV family* between the plant-specific *Arabidopsis thaliana, Solanum lycopersicum, Brassica juncea, Zea mays L.* and *Oryza sativa* (Figs. 1, 2, 3, and 4). In contrast, the *RRTF1* gene was conserved among all species with their sequence-specific motifs (Figs. 5 and 6). The phylogenetic tree demonstrated the molecular evolutionary link between the *RRTF1* genes in the *Arabidopsis thaliana, Solanum lycopersicum*, *Brassica juncea*, *Zea mays L. *and *Oryza sativa* (Fig. 7). Also, the phylogeny analysis demonstrated the particular clade represented the *AP2 family, ERF family, DREB sub-family* and *RAV family* (Fig. 8). Further, the *RRTF1* (*Redox responsive transcription factor 1*) expression is highly revealed in the flowering stage and minimal in the germinating period of the plants and observed abundant in flower, blade, hypocotyl, lateral root, and cotyledon.

**Table 3:** Summary of the GO annotation

Gene Id	Gene	Protein	Species
AT4G34410.1	*ERF109*	*ethylene-responsive transcription factor ERF109*	*Arabidopsis thaliana*
Solyc10g050970	*ERF109*	*ethylene-responsive transcription factor ERF109*	*Solanum lycopersicum*
Solyc01g108240	*ERF109*	*ethylene-responsive transcription factor ERF109*	*Solanum lycopersicum*
BjuB05g59080S	*ERF109*	*ethylene-responsive transcription factor ERF109*	*Brassica juncea*
BjuA03g05680S	*ERF109*	*ethylene-responsive transcription factor ERF109*	*Brassica juncea*
BjuA01g40180S	*ERF109*	*ethylene-responsive transcription factor ERF109*	*Brassica juncea*
BjuB02g73640S	*ERF109*	*ethylene-responsive transcription factor ERF109*	*Brassica juncea*
BjuA08g14310S	*ERF109*	*ethylene-responsive transcription factor ERF109*	*Brassica juncea*
BjuB03g42060S	*ERF109*	*ethylene-responsive transcription factor ERF109*	*Brassica juncea*
Zm00001eb100800	*ERF109*	*ethylene-responsive transcription factor ERF109*	*Zea mays L*
Zm00001eb038070	*ERF109*	*ethylene-responsive transcription factor ERF109*	*Zea mays L*
BGIOSGA030907	*ERF109*	*ethylene-responsive transcription factor ERF109*	*Oryza sativa*

Summary of the GO annotation of *RRTF1 (ERF109)* in all species

## Discussion

The study demonstrated the *Apetala 2/ethylene-responsive factor* family between *Arabidopsis thaliana, Solanum lycopersicum, Brassica juncea, Zea mays L.,* and *Oryza sativa*. The earlier empirical data suggested that Sakuma et al. (2002) reported 17, 121, and 6 genes in the *Apetala 2 family*, *ethylene-responsive factor family*, and *Related to ABI3 and VPI family* in a superior model plant of *Arabidopsis thaliana* respectively. Also, the collaboration of Nakano et al. (2006) suggested 18, 122, and 6 *AP2 domain*-associated isoforms in the *Apetala 2 family*, *ethylene-responsive factor family*, and *Related to the ABI3 family* in universal *Arabidopsis thaliana* and also compared with 139 specimens in the *ethylene-responsive factor family* of *Oryza sativa*. This study upgraded the total number of plant-specific genes in the *Apetala 2 (AP2) family*, *ethylene-responsive factor (ERF) family*, *DREB subfamily*, and *RAV (Related to the ABI3) family* of the crop-specific *Arabidopsis thaliana, Solanum lycopersicum, Brassica juncea, Zea mays L.* and *Oryza sativa* (Table 4). The *Brassica juncea* exhibited the highest number of specific hits, whereas *Oryza sativa* showed the lowest. So, the study forwarded the developmental genes in the *AP2 family* (*i.e.* flower, meristem, leaf, and embryo development). In contrast, the abiotic stress-responsive genes in the *ERF (ethylene-responsive factor) family* and *DREB subfamily* (*i.e*. low oxygen, freezing, drought, salt, oxidative, osmotic, heat, ABA ethylene, jasmonic acid, and abscisic acids) (Table 5). At last, the involvement of the *RAV family* in a brassinosteroid response were observed.

**Table 4:** Summary of the *AP2/ERF super-family*

**Classification**	***Arabidopsis thaliana***	***Solanum lycopersicum***	***Brassica juncea***	***Zea mays L***	***Oryza*** ***sativa***
*AP2 Family*	56	23	24	75	32
*ERF Family*	134	148	381	219	127
*DREB Family*	8	4	12	8	5
*RAV Family*	26	7	53	25	14
**Total**	**224**	**182**	**470**	**327**	**178**

Summary of the (a) *AP2 family* (b) *ERF family* (c) *DREB sub-family* and (d) *RAV family*

**Table 5:** Biological Function of genes in the* AP2/ERF family*

**Plant Species**	**Gene**	**Biological Function**
*Arabidopsis thaliana*	*TINY*	Negatively regulated development and Positively regulated abiotic stress
*Arabidopsis thaliana*	*RAP2.4*	Positively regulated light and ethylene and drought stress response
*Arabidopsis thaliana*	*ERF4*	Positively regulated ethylene and ABA response
*Arabidopsis thaliana*	*RAP 2.3*	Positively regulated low oxygen, oxidative, and osmotic stress response
*Arabidopsis thaliana*	*ERF53*	Positively regulated heat and ABA response
*Arabidopsis thaliana*	*ERF1*	Positively regulates salt, drought, and heat stress response
*Arabidopsis thaliana*	*DREB2* *A*	Positively regulated drought, salt, heat, and cold stress response
*Arabidopsis thaliana*	*ERF109*	Oxidative stress reponse (Redox response)
*Arabidopsis thaliana*	*RAP2.4*	Positively regulates light- and ethylene-mediated growth regulation
*Arabidopsis thaliana*	*ERF7*	Abscisic acid response
*Arabidopsis thaliana*	*WIN1*	Wax accumulation
*Arabidopsis thaliana*	*ABR1*	Abscisic acid response and sugar signaling
*Arabidopsis thaliana*	*AG*	Floral homeotic response
*Arabidopsis thaliana*	*RAV1*	Low temperature response

Biological functions of the *AP2/ERF family* in *Arabidopsis thaliana*

So, the *AP2/ERF super-family* is necessary to explore the *sub-families* involved during the growth and survival of crops. A comparative analysis of crops' genomes is mandatory for agriculture science and development. Also, the crops are manageable through agriculture biotechnology for research and development. In contrast, approximately 80% of crops produce in India. Those crops are economically beneficial around the globe.

## Conclusion

The ecosystem depends on a balance among flora and fauna. The living organisms build on the food cycle to manage the Eco-system. Ecologically, plants are the subject of survival organisms. In addition, the crops are vital for a healthy life span. The cultivation of crops proposes the knowledge of agriculture biotechnology. So, the perusal of crop genomes is necessary to observe stress and developmental-responsive genes in particular species. This study summarized genes in the *AP2/ERF super-family* in different crops-genomes. Those species-specific genes are necessary for the growth and survival of crops. Crops are economically valuable worldwide. Therefore, the study provided extensive knowledge of the agronomic, economic, and ecological traits and possibly other benefits of crops. Also, the documented data provide valuable information in plant databases for agriculture research and development.

## Methods

### Sequences and database

The current draft genome sequences of *Arabidopsis thaliana, Solanum lycopersicum*, *Brassica juncea*, *Zea mays L., *and *Oryza sativa* was download from the TAIR (https://www.arabidopsis.org/) and Ensemble (https://asia.ensembl.org/index.html) genome database. *The Arabidopsis Information Resource* (TAIR) database provides the option to attain the target sequence of model organisms of *Arabidopsis thaliana*. Also, SMART (http://smart.embl-heidelberg.de/) and Pfam (http://pfam.xfam.org/) retrieves to identify the particular domains in the *AP2/ERF family*.

### Standalone tools and gene ontology (GO) annotation

The HMMER algorithm executes by the MSA of the specific domain as a profile search. HMMER is a statistical algorithm that allows MSA (making multiple sequence alignment) of the particular domain as a profile search. It is an implemented practice of the probabilistic norm called the profile hidden Markov pattern. Standalone BLAST2 performs for homolog genes in both organisms. Also, BLAST2GO performs for the sequence accuracy of a specific transcription factor in the genome. BLAST2GO (BioBam) is a bioinformatics and statistical tool for high-throughput GO annotation of the novel sequence.

### Sequence domain, motif, and phylogeny

Multiple sequence alignment (MSA) systems was used to calculate the average match of the homologous sequences for the identities, similarities, and differences that appear. MSA of multiple hits sequences analysis done by a web-based application MultAlin (http://multalin.toulouse.inra.fr/multalin/) for identification and upgradation of the conserved domain. Also, the MEME suite is commonly known as a computational web-based tool for analysis and even discovery of sequence-specific motifs, so retrieve specific motifs via MEME suite (https://meme-suite.org/meme/). Finally, the perusal of the molecular evolutionary link between genes and their particular families in between *Arabidopsis thaliana, Solanum lycopersicum*, *Brassica juncea*, *Zea mays L.,* and *Oryza sativa*, performed MEGA7 for constructing a phylogenetic tree by *Neighbor-Joining Methods*.

## Data Availability

The data and material may be available on request through the corresponding author of Shouhartha Choudhury (Email Id: shouharthac@gmail.com). Gene Id 829591 TF Id: AT4G344110.1 https://www.arabidopsis.org/ https://www.arabidopsis.org/servlets/Search?type=general&search_action=detail&method=1&show_obsolete=F&name=ERF109&sub_type=gene&SEARCH_EXACT=4&SEARCH_CONTAINS=1 https://www.arabidopsis.org/servlets/TairObject?id=126772&type=locus http://plants.ensembl.org/index.html
